# Neuroprotective Effect of Curcumin on the Nigrostriatal Pathway in a 6-Hydroxydopmine-Induced Rat Model of Parkinson’s Disease is Mediated by α7-Nicotinic Receptors

**DOI:** 10.3390/ijms21197329

**Published:** 2020-10-03

**Authors:** Eslam El Nebrisi, Hayate Javed, Shreesh K Ojha, Murat Oz, Safa Shehab

**Affiliations:** 1 Department of Pharmacology, Dubai Medical College, Dubai Medical University, Dubai 20170, UAE; dr.eslam@dmcg.edu; 2 Department of Anatomy, College of Medicine and Health Sciences, United Arab Emirates University, Al-Ain PO BOX 17666, UAE; h.javed@uaeu.ac.ae; 3 Department of Pharmacology and Therapeutics, College of Medicine and Health Sciences, United Arab Emirates University, Al-Ain PO BOX 17666, UAE; shreeshojha@uaeu.ac.ae (S.K.O.); murat.oz@hsc.edu.kw (M.O.); 4 Department of Pharmacology and Therapeutics, College of Pharmacy, Kuwait University, Kuwait 24923, Kuwait

**Keywords:** Parkinson’s disease, 6-OHDA, α7-nAChR, curcumin, neuroprotection

## Abstract

Parkinson’s disease (PD) is a common neurodegenerative disorder, characterized by selective degeneration of dopaminergic nigrostriatal neurons. Most of the existing pharmacological approaches in PD consider replenishing striatal dopamine. It has been reported that activation of the cholinergic system has neuroprotective effects on dopaminergic neurons, and human α7-nicotinic acetylcholine receptor (α7-nAChR) stimulation may offer a potential therapeutic approach in PD. Our recent in-vitro studies demonstrated that curcumin causes significant potentiation of the function of α7-nAChRs expressed in *Xenopus* oocytes. In this study, we conducted in vivo experiments to assess the role of the α7-nAChR on the protective effects of curcumin in an animal model of PD. Intra-striatal injection of 6-hydroxydopmine (6-OHDA) was used to induce Parkinsonism in rats. Our results demonstrated that intragastric curcumin treatment (200 mg/kg) significantly improved the abnormal motor behavior and offered neuroprotection against the reduction of dopaminergic neurons, as determined by tyrosine hydroxylase (TH) immunoreactivity in the substantia nigra and caudoputamen. The intraperitoneal administration of the α7-nAChR-selective antagonist methyllycaconitine (1 µg/kg) reversed the neuroprotective effects of curcumin in terms of both animal behavior and TH immunoreactivity. In conclusion, this study demonstrates that curcumin has a neuroprotective effect in a 6-hydroxydopmine (6-OHDA) rat model of PD via an α7-nAChR-mediated mechanism.

## 1. Introduction

Parkinson’s disease (PD) is the second most common age-related slowly progressive neurodegenerative disease after Alzheimer’s disease (AD) [1]. It is prevalent in approximately 1% of the population over the age of 60 and 0.3% of people of all ages in industrialized countries [2]. Pathologically, the hallmark of the disease is the phosphorylation of the α-synuclein protein and formation of proteinaceous inclusions including Lewy bodies in neurons and Lewy neurites in axons and dendrites, as well as degeneration of dopaminergic nigrostriatal neurons [3,4]. The majority of cases are sporadic but about 10–15% of patients have a positive family history of PD [5]. Among a number of triggers, exposure to some environmental insults contributes to the degenerative changes observed in PD including mitochondrial dysfunction, oxidative stress, modifications in protein handling, adaptations in immune-modulators, and alterations to other molecular and cellular functions [6,7,8]. The motor signs and symptoms of PD, including bradykinesia, resting tremor, rigidity, and postural instability, begin to appear when 60–80% of the dopamine-producing neurons in the substantia nigra (SN) are defunctionalized [9]. Other central nervous system neurotransmitter systems are also affected to varying degrees, including cholinergic, γ-aminobutyric acid (GABA)-ergic, glutamatergic, tryptaminergic, noradrenergic, and adrenergic nerve cells that may exhibit similar damage to their cytoskeletons [10]. Being mainly a dysfunction of the brain’s dopaminergic system, Levodopa or L-dopa (L-3,4-dihydroxyphenylalanine), a prodrug of dopamine that enhances the intracerebral dopamine concentration, has, to date, been the gold standard treatment for PD. However, after several months to years of treatment, L-dopa induces dyskinesia in patients [11,12]. A crucial unmet demand in the management of PD is the discovery of new strategies that slow, stop, or even reverse the process of neurodegeneration. Several pieces of experimental evidence have revealed that the cholinergic system is a potential pharmacological target for the treatment of PD [13,14]. In addition, several studies have correlated the decline in specific nicotinic acetylcholine receptors (nAChRs) with certain pathological conditions, including neuropathic pain [15,16], autism [17], epilepsy [18], schizophrenia [19], AD [20], and PD [14].

Nicotinic receptors are members of a structurally related family of ligand-gated ion channels that include receptors for neurotransmitters such as 5-hydroxytryptamine (5-HT), γ-aminobutyric acid (GABA), and glycine [21,22]. Nicotinic acetylcholine receptors have a pentameric structure consisting of five transmembrane subunits around a central water-filled pore, which is selective for cations [22]. To date, 16 distinct nAChR subunits have been identified. The subunits are divided into two subgroups, α and β, of which five are expressed in muscle (α1, β1, γ, δ, and ε) and 11 in nervous tissue (α2–7, α9, α10, and β2–4) [21,23].

Among these subtypes, neuronal α7-nAChR, which forms a functional homomeric receptor, has received enormous pharmacological attention, due to its localization and function, as well as favorable features such as high calcium permeability (PCa/PNa ≈ 10), rapid activation and desensitization by agonists (millisecond scale) [24,25], and selective inhibition by α-bungarotoxin (α-Btx) and methyllycaconitine (MLA) [25,26]. Because of its simple organizational structure, which is relatively conserved in all vertebrate species, the α7 subunit has been of interest in the study of structure–function relationships [27]. The regional distribution of nAChR subtypes has been described to be in the temporal cortex, cerebellum, thalamus, striatum, and basal forebrain [23]. The alteration of cholinergic neurotransmission, either by genetic dysregulation or cholinergic denervation, has been demonstrated in various studies. Among the many natural products available for medicinal use, the polyphenolic ingredient of dietary turmeric (*Curcuma longa*), curcumin, has attracted attention due to its multiple benefits to patients with various diseases including PD [28].

Several studies have demonstrated that curcumin treatment significantly inhibits the toxin-induced loss of dopaminergic neurons in both cultured cells and animal models. Curcumin was found to exert neuroprotective effects mediating anti-oxidant, anti-inflammatory, and anti-apoptotic properties and improve neurological functions in various animal models of PD [29]. However, the receptor-based pharmacological mechanism of the molecule must be established to provide a rationale for the therapeutic targeting of α7-nAChRs for drug discovery and development of curcumin for pharmaceutical and clinical use. Using this approach, our recent in vitro data demonstrated that curcumin exerts significant potentiation of the action of α7-nAChRs expressed in *Xenopus* oocytes, acting as a type II positive allosteric modulator (PAM II) of α7-nAChRs [30,31].

The aim of the present study was to investigate the protective effects of curcumin in a 6-hydroxydopmine (6-OHDA)-induced animal model of PD and to determine whether α7-nAChRs are involved in mediating the effects of curcumin.

## 2. Results

The neuroprotective effects of curcumin given orally (200 mg/kg) 2 weeks pre- and post-surgery were determined by behavioral and morphological analyses. The assessment of motor function was performed 3 weeks after surgery. This was followed by brain sectioning and processing of the immunohistochemistry.

### 2.1. Apomorphine-Induced Rotation Test

**Figure 1:**
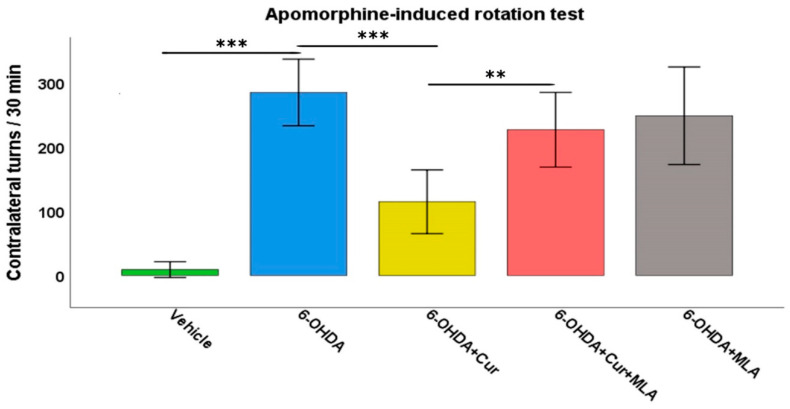
The motor performance of rats was assessed using an apomorphine-induced rotation test (0.25 mg/kg) expressed as full body turns over a period of 30 min. The vehicle group showed a normal turning response with no preference for a particular direction. The turning response significantly increased in 6-hydroxydopmine (6-OHDA)-injected rats (ANOVA, *p* ˂ 0.000 **). Curcumin pre- and post-treatment significantly reduced the turning response in comparison with the 6-OHDA group (ANOVA, *p* ˂ 0.000 ***) through an α7-acetylchoine receptor mediated mechanism. Administration of methyllycaconitine (MLA), an α7-receptor blocker, abolished the effect of curcumin (ANOVA, *p* ˂ 0.007 **) and the number of turns was comparable with that seen in the 6-OHDA group (ANOVA, not significant (NS)). MLA on its own had no effect, as indicated by the last group, for which the number of turns were comparable those seen in the 6-OHDA+Cur+MLA or 6-OHDA injected rats (ANOVA, NS).

Deficits in motor function were clearly observed in the 6-OHDA-treated rats compared with the vehicle-treated control group. The injection of apomorphine into these rats provoked a strong contralateral turning response with an average of 257.8 ± 23.4 turns/30 min in the 6-OHDA group, in comparison with the vehicle-treated group in which turns in both directions were almost negligible (8.9 ± 5.0 turns/30 min, ANOVA, *p* < 0.000). Statistical analyses demonstrated that curcumin treatment improved motor performance in the 6-OHDA+Cur group, as the turning response was significantly lower than that in the group administered with 6-OHDA (126.9 ± 23.8 turns/30 min, ANOVA, *p* < 0.000). However, the administration of the α7-nAChR blocker MLA almost nullified the neuroprotective effect of curcumin in the group treated with 6-OHDA+Cur+MLA compared with the 6-OHDA+Cur group (226.9 ± 23.8 turns/30 min, ANOVA, *p* < 0.007). The aim of having a 6-OHDA+MLA animal group was to investigate the effect of MLA per se. Therefore, the intraperitoneal (I.P.) injection of MLA preceded subcutaneous injection of apomorphine by 10 min in the 6-OHDA+MLA-treated group. The MLA-treated group did not exhibit any statistically significant differences compared with the 6-OHDA or 6-OHDA+Cur+MLA groups (231.7 ± 30.2 turns/30 min, ANOVA, not significant (NS) in both cases) (Figure 1). MLA itself did not have any effect on turning response, as indicated by the motor assessment performed before and after MLA injection (250.1 ± 39.4 compared to 231.7 ± 30.2 turns/30 min, Paired *t*-test, NS).

### 2.2. Morphological Analysis

#### 2.2.1. Effects on Tyrosine Hydroxylase-Positive Striatal Innervation

**Figure 2:**
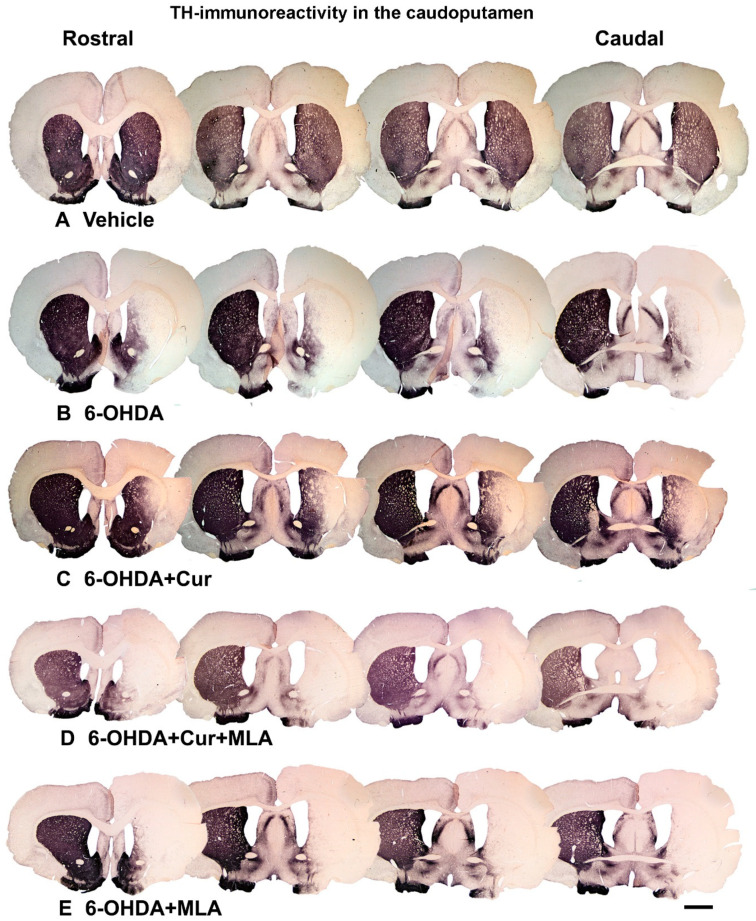
Coronal sections of the brain showing tyrosine hydroxylase (TH)-immunoreactive fibers in four approximately equally spaced rostro-caudal levels in the five animal groups included in the study. The injection of vehicle produced no significant effect on TH immunoreactivities (**A**); In comparison, the intra-striatal 6-OHDA lesion caused extensive reduction in the density of TH-immunoreactive fibers, mainly in the central and lateral parts of head and tail of the caudate-putamen, leaving the medial and ventral sectors partially intact (**B**); Curcumin treatment protected TH-positive fiber innervation to a large extent (**C**); However, 6-OHDA+Cur+MLA treatment reversed the protective effect of curcumin (**D**) but MLA treatment alone showed no significant difference from 6-OHDA or 6-OHDA+Cur+MLA (**E**). Scale bar = 2 mm.

The extent of dopamine denervation caused by the intra-striatal 6-OHDA injections was analyzed by tyrosine hydroxylase (TH)-immunohistochemistry in serial coronal sections throughout the rostro-caudal extent of the striatum. The quantitative assessment of striatal TH fiber density was carried out by conducting optical density measurements at four defined rostro-caudal levels, as shown in Figure 2.

**Figure 3:**
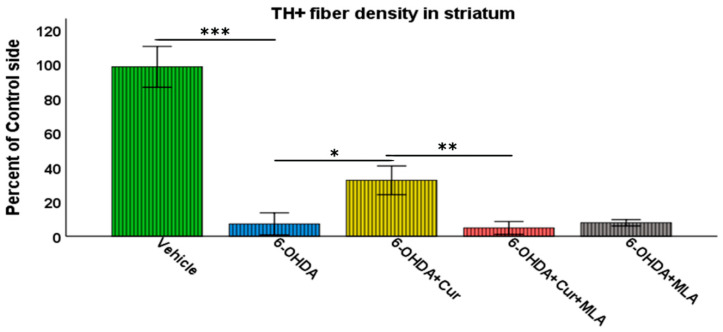
Striatal TH-immunoreactive fiber density expressed as a percentage of the fiber density on the lesioned side to the non-lesioned side. The density was measured at four rostro-caudal levels of the striatum: rostral (+1.56 mm), middle 1 (+0.72 mm), middle 2 (+0.12 mm), and caudal (−0.24 mm) relative to the bregma. The multiple intra-striatal 6-OHDA injections caused extensive damage to TH-positive fibers throughout the four levels of striatum compared with the vehicle-treated group (ANOVA, *p* ˂ 0.000 ***). The neuroprotective effect of curcumin, through an α7-receptor-mediated mechanism, was marked in the curcumin-treated group at all rostro-caudal levels of striatum in comparison with the 6-OHDA-injected group (ANOVA, *p* ˂ 0.011 *). MLA, an α7-receptor blocker, reversed the protective effect of curcumin in the 6-OHDA+Cur+MLA group in comparison with the 6-OHDA+Cur-treated group (ANOVA, *p* ˂ 0.005 ** at all levels). The MLA antagonist had no effect by itself, as indicated by the last group of animals (6-OHDA+MLA) and exhibited no statistical difference from the 6-OHDA+Cur+MLA group or 6-OHDA injected rats at all levels (ANOVA, NS). Values represent mean % of control side ± 2 SEM.

Vehicle-treated rats did not show significant differences between lesioned and non-lesioned sides throughout the four rostro-caudal levels of the striatum (98.29 ± 5.9/ANOVA). In contrast, the administration of multiple 6-OHDA injections resulted in an extensive reduction in the amount of TH immunoreactivity seen throughout the four-rostro-caudal levels of the striatum compared with the vehicle-treated group (7.14 ± 3.2/ANOVA, *p* ˂ 0.000). In the third group of rats, pre- and post-surgical curcumin treatment was administered orally on a daily basis for 4 weeks. Curcumin treatment had a restorative effect on TH-immunoreactive fibers compared with the group injected with 6-OHDA (32.46 ± 4.2/ANOVA, *p* ˂ 0.011). To investigate whether the effect of curcumin was mediated via α7-nAChRs, rats were given an I.P. injection of the selective α7-nAChR blocker MLA daily, prior to curcumin treatment. Compared with the level of neuroprotection seen in the 6-OHDA+Cur group, blocking α7-nAChRs was shown to reverse the neuroprotective effect of curcumin throughout the four levels of striatum in 6-OHDA+Cur+MLA-treated rats (4.81 ± 1.85/ANOVA, *p* ˂ 0.005), with no statistical difference from the 6-OHDA group (*p* ˃ 1.00). When the same amount of toxin was given to 6-OHDA+MLA animals, the MLA injection before motor assessment showed no effect on striatal innervation, compared with the 6-OHDA or 6-OHDA+Cur+MLA groups (7.79 ± 0.9/ANOVA, *p* ˃ 1.00) as shown in Figure 3.

#### 2.2.2. Effects on Tyrosine Hydroxylase-Positive Neurons in Substantia Nigra

The total number of SN TH-positive neurons was assessed bilaterally (using the left un-lesioned side as a control) in each animal using unbiased stereological analyses involving the optical fractionator principle. The counted region included the substantia nigra pars compacta (SNpc). The numbers of TH-positive neurons at three different levels of SN (caudal, middle, and rostral) were counted for each animal, to overcome several anatomical and technical confounding factors such as non-homogeneous distribution of dopaminergic neurons, variability between animals, and variability in immunohistochemical staining between batches of specimens. The intra-striatal injection of 6-OHDA caused a substantial loss of SN cells, while sparing TH-positive neurons in the ventral tegmental area (VTA). There was no significant difference bilaterally in the number of neurons in the vehicle-treated group at the three levels and in the analysis of the total surviving SN neurons (ANOVA, rostral: 100.3 ± 6.2, middle: 94.0 ± 6.6, caudal: 105.6 ± 5.0, total: 99.7 ± 2.5). However, on day 21 after 6-OHDA lesioning, the number of the TH-positive neurons was markedly decreased on the lesioned side compared with the vehicle-treated group at all rostro-caudal levels (ANOVA, rostral: 11.3 ± 2.9 (*p* ˂ 0.000), middle: 10.2 ± 2.0 (*p* ˂ 0.000), caudal: 8.9 ± 2.6 (*p* ˂ 0.000), total: 9.9 ± 1.9 (*p* ˂ 0.000)). Curcumin administration pre- and post-treatment had a neuroprotective effect and significantly restored 6-OHDA-induced damage to dopaminergic neurons in comparison with 6-OHDA-injected animals at the caudal, middle, rostral levels and in the analysis of total surviving SN neurons in the 6-OHDA+Cur-treated group (ANOVA, rostral: 30.2 ± 5.1 (*p* ˂ 0.003), middle: 39.2 ± 6.8 (*p* ˂ 0.000), caudal: 42.5 ± 4.6 (*p* ˂ 0.000), total: 32.0 ± 4.7 (*p* ˂ 0.000)). In contrast, blocking of α7-nAChRs with the I.P. injection of MLA prior to the oral administration of curcumin significantly reversed the neuroprotective effect of curcumin at all three levels in the 6-OHDA+Cur+MLA-treated group (ANOVA, rostral: 6.7 ± 0.9 (*p* ˂ 0.000), middle: 12.3 ± 2.6 (*p* ˂ 0.000), caudal: 12.9 ± 1.3 (*p* ˂ 0.000), total: 10.3 ± 1.4 (*p* ˂ 0.000)), supporting the suggestion that the neuroprotective effect of curcumin is mediated via α7-nAChRs.

**Figure 4:**
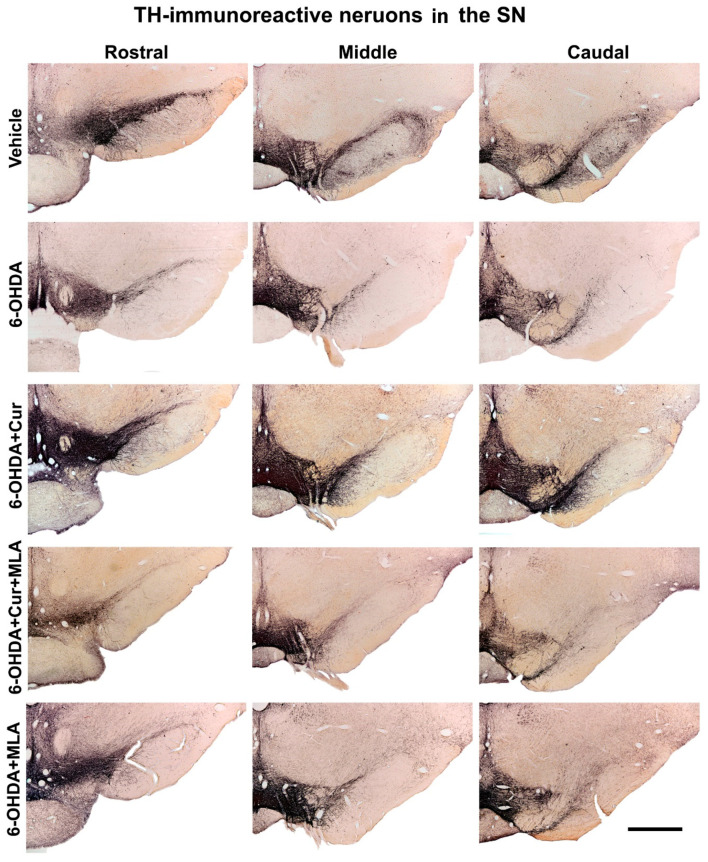
Photomicrographs of coronal sections of the midbrain showing TH immunoreactivity in three rostro-caudal levels of substantia nigra (SN) on the lesion sides. In comparison with the vehicle treatment, 6-OHDA-treated rats exhibited severe loss of TH-positive cells in the substantia nigra pars compacta (SNpc) at all levels. Curcumin-treated rats displayed significant improvements in cell survival in comparison with 6-OHDA-injected rats. MLA, an α7-receptor blocker, reversed the protective effect of curcumin in the 6-OHDA+Cur+MLA group. The MLA antagonist had no effect by itself, however, as indicated by the last animal group, 6-OHDA+MLA, which exhibited no statistical difference from the 6-OHDA+Cur+MLA group or 6-OHDA-injected rats. Scale bar = 1 mm.

**Figure 5:**
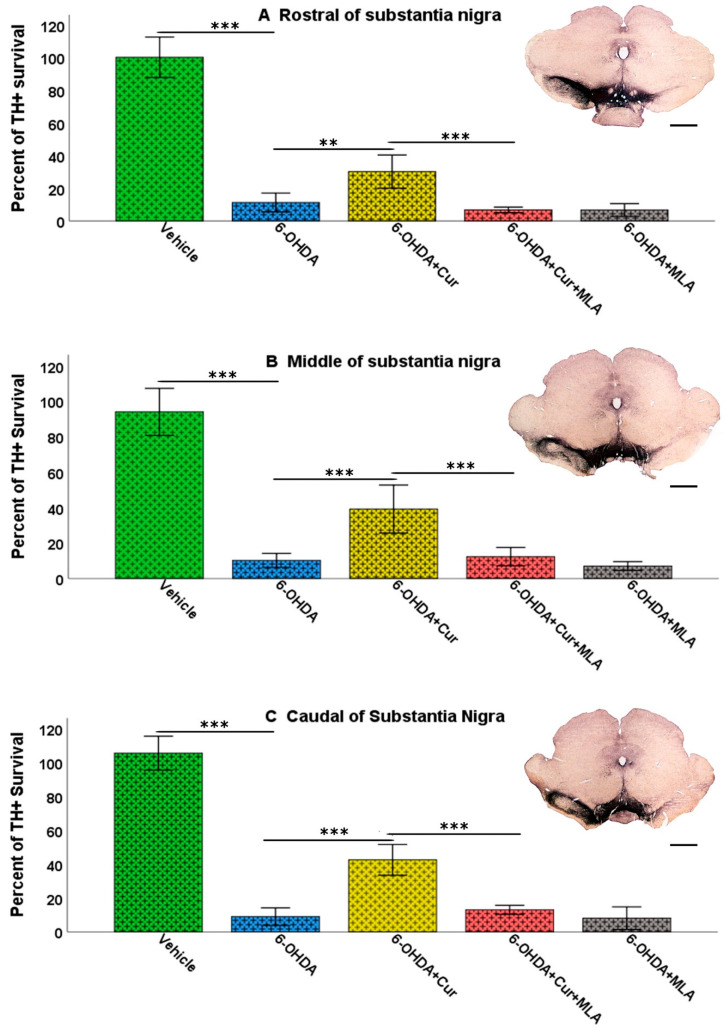
Stereological assessment of the total numbers of TH-positive cell bodies in the SNpc at all three levels: rostral, middle, and caudal. Multiple intra-striatal 6-OHDA injections caused a dramatic loss of TH-positive neurons in the SNpc compared with the vehicle-treated animals (ANOVA, rostral, middle, caudal, and total, *p* ˂ 0.000 ***, at all levels). The neuroprotective effect of curcumin, through an α7-receptor mediated mechanism, was marked in the group treated with curcumin at all rostro-caudal levels: rostral (**A**); middle (**B**); and caudal (**C**) in comparison with the 6-OHDA-treated group (ANOVA, rostral *p* ˂ 0.003 **, middle *p* ˂ 0.000 ***, and caudal *p* ˂ 0.000 ***). MLA, an α7-receptor blocker, reversed the protective effect of curcumin in the 6-OHDA+Cur+MLA group (ANOVA, *p* ˂ 0.000 *** at all levels). The antagonist MLA had no effect on its own, as indicated by the results from the 6-OHDA+MLA group, which exhibited no statistical difference from the 6-OHDA+Cur+MLA group or 6-OHDA-injected rats (ANOVA, NS, at all levels). Scale bar = 1 mm.

The neuronal count of the SN in the 6-OHDA+MLA group was comparable to that in the 6-OHDA+Cur+MLA or 6-OHDA groups with no statistical difference between the three groups at all levels (ANOVA, rostral: 6.7 ± 1.9, middle: 7.0 ± 1.2, caudal: 8.0 ± 3.3, total: 7.1 ± 2.0, *p* ˃ 1.0 at all levels). No changes at the cellular level were expected in this group and the main purpose of this category was to test behavioral variation caused by the administration of MLA. It is notable that, in all sections, both the morphology and number of TH-positive neurons in the SN on the un-lesioned left side remained unchanged (Figure 4 and Figure 5).

#### 2.2.3. Relationship between Morphological and Behavioral Parameters

**Figure 6:**
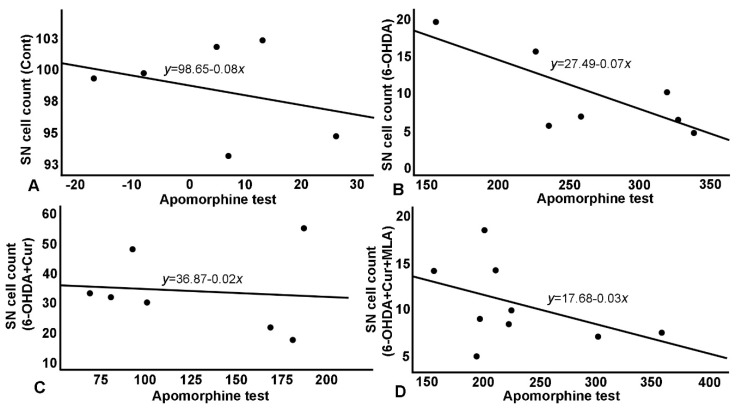
Relationship between apomorphine-induced rotation test and SN cell count. (**A**) A negative correlation in control group, with normal cell count (~100%) as reflected on number of rotation; (**B**) A negative correlation in 6-OHDA group with a steeper curve and a much-reduced number of surviving cells. 6-OHDA injected rats showed severe loss of TH+ve cells in the SN which affected rats’ performance in apomorphine test and in the shallower and less steep negative correlation; (**C**) Negative correlation in 6-OHDA+Cur group, where curcumin showed significant improvement in cells survival which was reflected significantly on apomorphine test; (**D**) Correlation in 6-OHDA+Cur+MLA group, MLA, an α7-receptor blocker, reversed the protective effect of curcumin in 6-OHDA+Cur+MLA group, and consequently apomorphine test results were comparable to 6-OHDA injected group.

**Figure 7:**
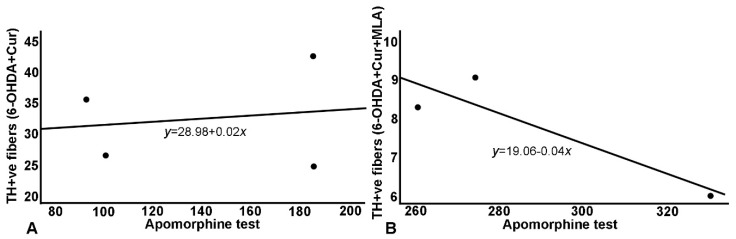
Relationship between apomorphine-induced rotation and striatal TH+ve fiber density: a similar relationship has been demonstrated with the same blunting effect on treatment as the effects seen with the SN cell count. (**A**) Correlation in 6-OHDA+Cur group, where curcumin administration improved striatal fiber density; (**B**) Concurrent MLA administration abrogated the effect of curcumin in 6-OHDA+Cur+MLA group.

As shown in Table 1 and Figure 6 and Figure 7, the TH-positive cell numbers in SN (expressed as percentage of control side) were negatively correlated with all other parameters (correlation coefficient R ≈ 0.30–0.76); distinguished correlations were observed (Figure 6). Similarly, striatal TH-positive optical fiber density also subtly correlated with the rats’ motor assessment scores (correlation coefficient > 0.7) in most cases (Figure 7).

## 3. Discussion

This study demonstrates that orally administered curcumin preserves the integrity of the nigrostriatal dopaminergic system, specifically cell bodies in SNpc, striatal terminals, and motor behavior in a 6-OHDA animal model of PD. Furthermore, the results indicate that the neuroprotective effects of curcumin are mediated by α7-nAChRs.

We assessed motor function using an apomorphine-induced rotation test, a gold standard assessment of unilateral 6-OHDA lesions. Stereotaxic injections of 6-OHDA induce contralateral rotations only in rats with severe dopamine (DA) depletion [32]. This test is considered reliable, objective, and closely related to the degree of nigrostriatal dysfunction, as well as DA depletion [33]. Previous studies have demonstrated that the intra-striatal injection of 6-OHDA causes retrograde degeneration of SN dopaminergic neurons, resulting in the depletion of striatal dopamine [34]. In agreement with these reports, we observed that, 21 days after the administration of 6-OHDA, rats exhibited significant impairment in motor function.

In addition to the restoration of motor function, preservation of the nigrostriatal neuronal pathway is considered to be an important neuroprotective mechanism required of any pharmacological agent. As curcumin treatment significantly decreased the abnormal turning response in rats administered with 6-OHDA, we investigated the neuroprotective effects of the molecule on the loss of dopaminergic neurons. An unbiased stereological technique was used to determine the total number of TH-positive neurons in the SNpc of both hemispheres. Curcumin treatment significantly reduced the 6-OHDA-induced degenerative effect on SNpc dopaminergic neuronal cells, indicating that it has a neuroprotective effect on 6-OHDA-induced damage to DA neurons. Furthermore, the results demonstrated that the neuroprotective effects of curcumin are mediated via α7-nAChRs, as seen in the 6-OHDA+Cur+MLA group. In these animals, the number of TH-positive neurons in the SN was comparable to that found in the group treated only with 6-OHDA, with no significant difference found between the groups. The denervated TH-positive striatal fibers reflected the restorative effect of curcumin distinctly, as curcumin significantly protected the striatal fibers from the 6-OHDA toxin through an α7-nAChR-mediated mechanism, as indicated by the abolished effect of curcumin following the administration of MLA to animals in the 6-OHDA+Cur+MLA group.

Several studies have demonstrated that curcumin treatment significantly reduces the toxin-induced loss of dopaminergic neurons in both cultured cells and animal models. Curcumin has, in fact, been shown to reduce 6-OHDA-induced neurotoxicity in a human dopaminergic cell line (SH-SY5Y) via attenuation of reactive oxygen species production, p53 phosphorylation, and reduction of the Bax/Bcl-2 ratio [35]. Moreover, curcumin was found to protect dopaminergic neurons from 6-OHDA-induced toxicity via restoration of striatal dopamine, dopamine metabolite, dihydroxyphenylacetic acid, and homovanillic acid in a 6-OHDA model of PD [36] In these studies, the neuroprotective action of curcumin may be mediated by a reduction of oxidative stress due to its iron-chelating property [37] or via the Wnt/β-catenin signaling pathway [38] or modulating bFGF/NGF/TrkA/Hsp70 expressions in the SN [39].

Additionally, in a mouse model of 1-methyl-4-phenyl-1,2,3,6-tetrahydropyridine (MPTP)-induced PD, curcumin and its metabolite tetrahydrocurcumin (ThC), considerably reduced monoamine oxidase type B (MAO-B)-induced neurotoxicity [40]. Furthermore, a recent systematic literature review involving 13 studies of different PD animal models that took place between 2005 and 2014 emphasized the anti-oxidant, anti-inflammatory, and anti-apoptotic properties of curcumin, proving its neuroprotective activity and ability to improve neurological functions in different animal models of PD [29]. In addition, it is not known whether curcumin interferes with 6-OHDA uptake into dopamine neurons. If so, this suggests another mechanism underlying the possible involvement of curcumin in the reduction of TH neuronal loss.

Previously, we demonstrated that curcumin significantly decreases the desensitization of α7-nAChRs by acting as a type II PAM [30] and potentiates the function of these receptors [31]. Moreover, α7-nAChR PAMs are reported to exhibit beneficial effects in animal models of PD [41,42,43]. In line with these reports, the results of our study indicate that α7-nAChRs play a role in mediating the neuroprotective effects of curcumin in a 6-OHDA-induced rat model of PD.

Nicotinic acetylcholine receptors are principal modulators of neuronal excitability throughout the central nervous system. Presynaptic nAChRs influence the release of neurotransmitters, while postsynaptic nAChRs participate in fast postsynaptic neurotransmission as an excitatory input in the hippocampus and subcortical areas including the VTA [44,45]. In an earlier study, the levels of striatal TH, dopamine transporters, vesicular monoamine transporters, dopamine and nicotinic receptors were found to be greater in nicotine-treated MPTP-lesioned Parkinsonian animals than in lesioned animals not receiving nicotine [46]. Several in vitro studies using primary cultures from different brain regions such as striatal, nigral, cortical, or neuronal cell lines have demonstrated that pretreatment with nAChR agonists offers neuroprotective activity against toxic insults via α7- or α4β2*-nAChR-mediated mechanisms [47,48,49,50,51]. Thus, compounds that can modulate α7-nAChRs may have a neuroprotective effect against 6-OHDA-induced toxicity, which leads to dopaminergic neuronal loss.

Modulation of dopamine release by the cholinergic system has been a highlight of many studies. Dopamine input to the striatum arises from midbrain dopamine neurons located in the VTA and SNpc, which innervate the ventral striatum (nucleus accumbens) and dorsal striatum (caudate-putamen) [52,53]. The striatum is innervated by a high density of axonal varicosities of dopaminergic nerves forming dopaminergic synapses [54,55]. The cholinergic striatal interneurons are the primary source of cholinergic input to the striatum, containing both muscarinic and nicotinic receptors [56,57]. Although this study suggests the central effects of MLA in the brain, its action via peripheral tissues cannot be ruled out. α7-nAchRs have been implicated in various immunological processes including the modulation of inflammation in different pathologic conditions [58] which have a crucial role in the “cholinergic anti-inflammatory pathway” [59]. It has been demonstrated that α7nAChRs are expressed by macrophages, monocytes, neutrophils, T cells, B cells, and dendritic cells and mediate anti-inflammatory responses when activated on these cells [60]. In addition, the α7nAChRs mediates the attenuation of cytokine release which can be blocked by α7 selective antagonists and are absent in cells from α7 knockout mice [60].

Curcumin is a naturally occurring compound that has been safety tested over time in humans and has negligible toxicity [61]. Several clinical trials have employed curcumin for the treatment of neurodegenerative disorders (e.g., AD and mild cognitive impairment). Dadhaniya et al. reported that the LD_50_ of curcumin in rats and mice was greater than 2000 mg/kg body weight, after 90 days of treatment [62]. Indeed, the dose used in present study (200 mg/kg orally) is equivalent to 2 g/day in humans, which is very close to the recommended dose suggested in an earlier report [63]. In agreement with several previous studies, in which curcumin and its derivatives have been shown to improve motor, cellular, and biochemical alterations in experimental models of PD [34,64,65,66], the findings of the present study clearly demonstrate a novel pharmacological mechanism for curcumin in neuroprotection mediated by α7-nAChRs.

## 4. Materials and Methods

### 4.1. Animals

Male Wistar rats weighing between 220 and 250 g at the beginning of the study were used. A maximum of four rats were housed in a large cage with free access to rat chow and water under a 12:12 h light/dark cycle at room temperature (22 °C). All experimental procedures were approved by the Animal Ethics Committee of the College of Medicine and Health Sciences, United Arab Emirates University and were performed in accordance with the guidelines of the European Communities Council directive of 24 November 1986 (86/609/EEC).

**Figure 8:**
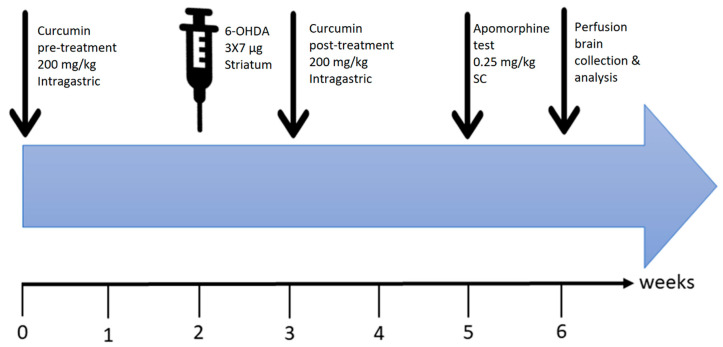
Time course of the experiments. Curcumin (200 mg/kg, per oral (P.O.))/MLA (1 µg/g, intraperitoneal (I.P.)) pretreatment was started 2 weeks before surgery. Intra-striatal injection of 6-OHDA was performed after week 2. This was followed by one-week of recovery to ensure head wound healing and allow proper animal handling for oral curcumin administration, which was continued for 2 weeks post-recovery. At the end of the 3-week total treatment period, an apomorphine-induced rotation test was conducted. Finally, animals were sacrificed, and the brains were collected for processing and data analysis.

The plan of the experiments is summarized in Figure 8. The rats were randomly assigned to one of five groups: (1) Vehicle-treated group (ascorbic acid was injected into the right striatum and the animals received daily oral gavage carboxymethyl cellulose (*n* = 7); (2) 6-OHDA-treated group (6-OHDA was injected into right striatum (*n* = 8)); (3) 6-OHDA+Cur group (given intragastric oral gavage curcumin (200 mg/kg) once a day for 4 weeks in total (2 weeks before and 2 weeks after surgery), with 6-OHDA lesions at the end of week 2 of curcumin pretreatment (*n* = 8)); (4) 6-OHDA+Cur+MLA group (the same as group 3, with the addition of an intraperitoneal (I.P.) injection of MLA 10 min before curcumin administration (*n* = 9)); and (5) 6-OHDA+MLA group (similar to group 2 with the addition of an I.P. injection of MLA 10 min before apomorphine-induced rotation testing (*n* = 8)).

### 4.2. Chemicals and Drugs

6-hydroxydopamine hydrochloride, apomorphine hydrochloride, and curcumin, were purchased from Sigma-Aldrich (Sigma Chemicals Co.; St. Louis, MO, USA) and MLA was purchased from Abcam, Cambridge, MA, USA. 6-OHDA-HCl, with purity ≥ 97%, was dissolved in ice-cold 0.01% ascorbic acid in 0.9% normal saline and used within 2 h of preparation. Apomorphine hydrochloride, with purity ≥98.5%, was dissolved in 0.01% ascorbic acid in 0.9% normal saline and prepared freshly before use. Curcumin was suspended in 0.5% sodium carboxy methylcellulose (CMC) and 50 µL of NaOH (10 M) and prepared freshly on a daily basis for dosing. MLA was dissolved in normal saline and preserved at 4 °C.

### 4.3. Surgical Procedure

All rats were deeply anesthetized using an I.P. injection of an equal mixture of ketamine hydrochloride (80 mg/kg, Troy Laboratory PTY Limited Glendenning, NSW, Australia) and xylazine hydrochloride (20 mg/kg, Troy Laboratory PTY Limited Glendenning, NSW, Australia). To lesion the nigrostriatal pathway, the heads of the animals were shaved and placed into a Stoelting stereotaxic frame and a unilateral hole on the right side of the skull was made as described before [67], aiming at the striatum. The rats received unilateral injections of 6-OHDA at three distinct locations within the caudate-putamen (7 µg in each site). Three intra-striatal 6-OHDA injections were performed using a pulled glass micropipette with an outer diameter of approximately 50 µm. Seven µg of 6-OHDA (dissolved in 2 µL) were injected at each site, using the following coordinates: antro-posterior (AP), +1.0, −0.1, −1.2; ML, −3.0, −3.7, −4.5; dorso-ventral (DV), −5.0, −5.0, −5.0. The tooth bar of the stereotaxic frame was fixed at 0.0 relative to the bregma [68]. 6-OHDA was injected at a rate of 1 µL/min and the injection micropipette was left in place for an additional 3 min to prevent backflow. All rats were allowed to recover for 3 weeks before behavioral testing. The vehicle-treated group received equivalent volumes of solvent (0.01% ascorbic acid) instead of the toxin.

### 4.4. Apomorphine-Induced Rotational Behavior

Three weeks after surgery, drug-induced rotational behavior was monitored in a rounded bowl (42 cm wide at the top and 22 cm deep) [69]. The animals were injected with apomorphine hydrochloride (0.25 mg/kg) dissolved in 0.01% ascorbic acid in normal saline [70] subcutaneously, or ascorbic acid (0.01%) alone for the vehicle-treated group, and rotational asymmetry was monitored. Immediately after vehicle or drug injections, the animals were allowed to acclimatize in the bowl for 5 min [71]. Turns of 360° in the clockwise and counter-clockwise directions were continuously recorded for 30 min. The net rotational asymmetry score was expressed as full body turns/minute. The direction contralateral to the lesion was considered positive. The rats that exhibited 5–7 full turns/minute were included in the study as Parkinsonian animals [72,73]. All rotational data were expressed as the mean of the full turns per minute ± 2 SEM. The data were expressed as the net (contralateral minus ipsilateral turns) rotations/minute. A single rat from each of these groups (6-OHDA-treated and 6-OHDA+MLA) did not pass the rotational asymmetry; thus, they were excluded from the analysis.

### 4.5. Histological Studies

The animals were euthanized with an overdose of 25% urethane (2 mL/200 g of animal weight) by I.P. injection and perfused transcardially through the ascending aorta with 50 mL of phosphate buffered saline (PBS), followed by 500 mL of 4% paraformaldehyde in phosphate buffer (PB, 0.1 M, pH 7.4) over 20 min. The brains were removed, post-fixed in the same fixative solution for 4 h, stored in 30% sucrose, and kept until they had sunk, before being sectioned using a cryostat. Coronal sections of the brain (40 µm thickness) at the striatum and SN were collected serially and processed for detection of tyrosine hydroxylase (TH) immunoreactivity using the avidin–biotin complex method of immunohistochemistry, as previously described [74,75].

### 4.6. Immunohistochemical Staining for Tyrosine Hydroxylase

Immunohistochemical staining was performed on free-floating sections. Briefly, the brain sections were rinsed three times with PBS solution followed by incubation in a 50% ethanol solution for 30 min to increase the penetration of the antibodies [76]. After rinsing in PBS, the sections were incubated overnight at room temperature in primary antibody solution containing 1% bovine serum albumin in PBS containing Triton-X-100. The primary antibodies used for immunohistochemical staining were raised in rabbit against TH, as a marker for dopaminergic neurons (AB152, Millipore; Burlington, MA, USA, at 1:1000 dilution). On the second day, the sections were rinsed in PBS and then incubated with biotinylated goat anti-rabbit IgG (Jackson Immunoresearch, West Grove, PA, USA, 1:500) for 1 h, then in extravidin–peroxidase conjugate (Sigma, 1:1000) for another hour. After rinsing, to visualize TH-positive immunoreactivity, the sections were incubated for 5–8 min in a solution of 25 mg diaminobenzidine (DAB) in 50 mL 0.1 M phosphate buffer (PB, pH 7.4) with 7.5 μL hydrogen peroxide (30%), and 1 mL nickel chloride (3.5%) was added to intensify the reaction. Finally, the sections were rinsed in PB and mounted on gelatin-coated slides. After drying in air, the sections were dehydrated in graded alcohol, cleared in xylene, and mounted with DPX mounting media. All antibodies were diluted in PBS containing 0.3% Triton-X-100.

### 4.7. Measurement of Striatal Fiber Density

Images of striatal TH-positive immunoreactive fibers were captured using a Nikon microscope (4.30 version, Nikon Corporation, Tokyo, Japan) equipped with a DS-Ri2 camera. The optical densitometry of TH-positive stained striatal terminals was measured using ImageJ software (1.8.0_112, NIH, Bethesda, MD, USA). The average optical density over the entire area of the striatum for four striatal sections in each animal was quantified: rostral (+1.56 mm), middle 1 (+0.72 mm), middle 2 (+0.12 mm), and caudal (−0.24 mm) in relation to the bregma in each animal. To compensate for differences in background coloring between the slides, the optical density was subtracted from values obtained in the cortex for each sample. The data were expressed as the percentage of fiber density on the lesioned side to the non-lesioned side as previously described [77,78,79,80].

### 4.8. Stereological Analysis: Substantia Nigra Cell Counts

The total number of TH-positive neurons in the substantia nigra pars compacta (SNpc) in both hemispheres was estimated by unbiased stereology using an optical fractionator [81], Stereo Investigator (Micro Bright Field, Colchester, VT, USA). The final number of animals included in the stereological analysis was as follows: Vehicle-treated = 7 (7), 6-OHDA = 8 (9), 6-OHDA+curcumin (Cur) = 8 (8), 6-OHDA+Cur+MLA = 8 (8), and 6-OHDA+MLA = 8 (8) (numbers in parentheses represent the number of animals that were used for behavioral testing). To estimate the number of TH-positive cells in the SNpc, the borders defining the SNpc were delineated using a low power objective lens (5×; S Plan) in referral to anatomical morphology. Counting was performed using a 63× Plan-Apo oil objective to generate counting areas of 106 × 106 µm. A counting frame (3184 µm^2^) was placed randomly on the first counting area and systematically moved through all counting areas. The section thickness was estimated to be 25 ± 5 µm, after dehydration and cover slipping, in different animals. Guard volumes of 2 µm were excluded from each surface to avoid the problem of lost caps. The sampling interval in the X–Y axis was adjusted so that at least 100 cells were counted for each region of interest. The coefficient of error due to the estimation was calculated according to [82] and values < 0.10 were accepted. For each animal, the number of TH-positive cells was measured in three sections at three different rostro-caudal levels of SN, according to the atlas of Paxinos and Watson (2005): (i) AP−4.8; (ii) AP−5.6; (iii) AP−6.2, relative to the bregma [75]. The data represent the percentage of surviving nigral TH-positive neurons per level, as well as the total survival analysis of the three rostro-caudal levels of the lesioned (right) side in comparison with the intact (left) side.

### 4.9. Data Analysis

One-way ANOVA with a post hoc Bonferroni test was used to assess the statistical significance of differences in behavioral tests and cell numbers between groups. Paired *t*-tests were used to compare apomorphine-induced rotation with and without MLA post-injection. The results were expressed as means ± SEM. The relationship between the behavioral and morphological parameters was assessed using Spearman’s rank correlation and simple linear regression analysis to analyze the log-transformed data.

## 5. Conclusions

The findings of the present study demonstrate that curcumin improves the survival of TH striatal fibers and neurons in SNpc and diminishes abnormal turning behavior in a 6-OHDA-induced rat model of PD. In the present study, abrogation of the protective effects of curcumin by pretreatment with MLA, an α7-nAChR-selective antagonist, clearly demonstrates that neuroprotective effects of curcumin are mediated by α7-nAChRs. Our findings clearly demonstrate that α7-nAChRs may be an attractive therapeutic target for PD and curcumin appears to be the first agent of natural origin to modulate α7-nAChRs in PD. Integrating our earlier observation that curcumin acts as a type II PAM of α7-nAChRs and potentiates receptor function by significantly decreasing desensitization, it is apparent that the PAM action of curcumin on α7-nAChRs exerts beneficial effects in mediating neuroprotective effects. With the time-tested safety and neuroprotective efficacy of curcumin and preliminary clinical success of agents targeting nicotinic receptors in PD, curcumin may be an attractive natural candidate for further investigation and development in the quest for PD therapeutics.

## Figures and Tables

**Table 1:** Correlation between the number of TH-positive cells in the SN and striatal TH-positive fiber density, and performance of animals in different study groups in the apomorphine-induced rotation test, as assessed by simple linear regression analysis, expressed as *R* and adjusted *R*^2^ values.

Groups	TH+ Cell Number in SN		TH+ Fiber Density in Striatum	
	*R*	*R* ^2^	*R*	*R* ^2^
Control	−0.313	−0.128	−0.697	−0.228
6OHDA	−0.763	−0.499	0.972	−0.888
6OHDA+Cur	0.092	0.190	−0.153	0.465
6OHDA+Cur+MLA	−0.450	−0.089	−0.476	−0.546
6OHDA+MLA	−0.449	−0.069	−0.910	−0.565

## Abbreviations

α7-nAChR	α7-nicotinic acetylcholine receptor
6-OHDA	6-hydroxydopamine
AD	Alzheimer’s disease
AP	Antro-posterior
Cur	Curcumin
DA	Dopamine
DV	Dorso-ventral
I.P.	Intraperitoneal
LD50	Lethal dose; 50%
MAO-B	monoamine oxidase type B
ML	Mediolateral
MLA	Methyllycaconitine
MPTP	1-methyl-4-phenyl-1,2,3,6-tetrahydropyridine
NaOH	Sodium hydroxide
NS	Not significant
SN	Substantia nigra
SNpc	Substantia nigra pars compacta
PAM	Positive allosteric modulator
PD	Parkinson’s disease
P.O	per oral
TH	Tyrosine hydroxylase
